# Growth conditions, physiological properties, and selection of optimal parameters of biodegradation of anticancer drug daunomycin in industrial effluents by *Bjerkandera adusta* CCBAS930

**DOI:** 10.1007/s10123-019-00102-3

**Published:** 2019-11-12

**Authors:** Teresa Korniłłowicz-Kowalska, Kamila Rybczyńska-Tkaczyk

**Affiliations:** Department of Environmental Microbiology, Laboratory of Mycology, The University of Life Sciences, Leszczyńskiego Street 7, 20-069 Lublin, Poland

**Keywords:** Anamorphic *Bjerkandera adusta* strain, Growth condition, Physiological properties, Biodegradation, Daunomycin

## Abstract

The study characterizes the anamorphic *Bjerkandera adusta* strain CCBAS 930, including growth conditions, physiological properties, and enzymatic activities related to basic metabolism and specific properties coupled with the fungal secondary metabolism. It was established that the fungus grows in a wide pH range (3.5–7.5), up to 3% of salt concentration and a temperature of 5–30 °C. Media rich in natural organic components (potato, maize extracts, whey) are optimal for biomass propagation. Minimal media, containing mineral salts and glucose as well as static growth conditions, are required to obtain idiophasic mycelium, equivalent to the secondary metabolism of the fungus. Of the 7 complex C, N, and energy sources tested, the strain did not utilize only fibrous cellulose. Lipolytic activity reached the highest values of the enzymatic activities corresponding to those capabilities. The specific properties of strain *B. adusta* CCBAS 930 determined by the production of HRP-like peroxidase were related to the decolorization and biodegradation of anthraquinone derivative daunomycin. The decolorization of 30% of daunomycin effluents occurred most rapidly in iso-osmotic medium and non-enriched with nitrogen, containing 0.25% glucose, pH = 5.0–6.0, and 25–30 °C. In agitated cultures, the strain decolorized solutions of daunomycin by biosorption, which coincided with the inhibition of aerial mycelium production and HRP-like biosynthesis. Based on knowledge, potential and real possibilities of using the strain in bioremediation of colored industrial sewage were discussed.

## Introduction

Daunomycin is a glycoside containing an amino sugar (glucosamine) and 3 anthracyclic rings (anthraquinonic compound) and a metoxylic group attached to the first ring. The aromatic component is responsible for biological activity and at the same time for the color (red pigment). The cytotoxic mechanism of action of daunomycin—as an inhibitor of DNA biosynthesis—involves the intercalation of this antibiotic into a newly synthesized polynucleotide chain, which leads to replication suppression (Zhang et al. 2013). Anthracycline antibiotics are obtained from the mycelium of *Streptomyces peucetius* or by chemical synthesis. Currently, the main producers of cytostatic drugs are China and India (50%), the USA (28%), Europe (15%), and Japan (12%) (Zhang et al. 2013). Recent years have seen an increase in the amount of pharmaceuticals and their metabolites in wastewater, particularly anti-tumor drugs (Lenz et al. 2007). Depending on the country, the level of anthracycline antibiotics in surface water ranges from ng/L to μg/L (Kolpin et al. 2002; Ashton et al. 2004; Lenz et al. 2007; Mompelat et al. 2009; Negreira et al. 2014).

These effluents cannot be introduced into general wastewater treatment plants without prior decolorization and detoxification, carried out by the chlorination method (Korniłłowicz-Kowalska et al. 2006a). Due to the carcinogenic, mutagenic, and teratogenic properties of cytostatic drugs, their presence in wastewater and surface water poses a serious threat to the aquatic environment (Kümmerer 2001, 2010; Zhang et al. 2013). Residues of these drugs can enter the surface water and groundwater, endangering both aquatic organisms and human health. Moreover, anthracycline antibiotics easily undergo single-electron reduction, generating semiquinone radicals and superoxide anion radicals. This initiates a cascade of free radical reactions whose products are highly toxic hydrogen peroxide and hydroxyl radical. Due to the universal character of the free radical mechanism of action of anthracyclines, all cells are exposed to the harmful effects of the radicals generated (Minotti et al. 2004).

Currently, wastewater containing pharmaceuticals is treated using physico-chemical methods: osmosis, ozonation, adsorption, membrane filtration and electrolysis (Gholami et al. 2012; Ikehata et al. 2007; Kümmerer 2009; Zhang et al. 2013). In recent years, biological methods using microorganisms to remove contaminants are gaining in importance, supplementing or presenting an alternative to conventional methods. Particularly promising in this regard are white-rot fungi (*Basidiomycetes*). White-rot fungi, and among them, species such as *Phanerochaete chrysosporium*, *Trametes versicolor*, *Pleurotus ostreatus*, and *Bjerkandera adusta* have been the subject of research as potential bioremediation factors for many years, especially in the context of environment purification from aromatic compounds. These compounds, and among them PAHs (polycyclic aromatic hydrocarbons), phenols, chlorophenols, azo, triarylmethane, and antraquinone dyes, post-industry lignins (modified lignins), such as Kraft lignin, lignosulfonates, chlorolignins, and pharmaceuticals are characterized by high toxicity, accumulation in the environment, and general low biodegradability.

Biological methods, consisting mainly in the use of microorganisms capable of degrading these compounds, are an alternative or a supplement to physico-chemical decolorization methods of colored industrial wastewater. Search and acquisition of highly efficient microorganisms that degrade a wide spectrum of colored contaminants with an aromatic structure remain an open issue. These compounds, due to their toxicity, cannot be directly introduced into general sewage treatment plants, e.g., municipal sewage, because they pose a threat to consortia of activated sludge microorganisms. This involves the necessity of their prior inactivation. Intensive coloring of wastewater from industries such as the pulp and paper industry or textile industry is also a problem. This sewage must be decolorized before introducing it into surface waters. Water colorization is a threat to biological life in this environment. This is mainly caused by a decrease in the inflow of light, which inhibits the development of phototrophs and, as a consequence, disturbance of the biological balance of waters characterized by excessive development of heterotrophic microorganisms, which exhaust oxygen, and hindering the existence of water micro- and macrofauna requiring oxygen for respiration.

*Bjerkandera adusta* (Willd. Ex.Fr) P. Karst (*Basidiomycota*, *Homobasidiales*, *Polyporales*, *Hapalopilaceae*) is one of the more common fungi in the environment causing white-rot, mainly of deciduous trees, especially beech, hornbeam, oak, and birch, but also some conifers, e.g., spruce. In addition to the perfect stage occurring in the forests (teleomorph), we also know about the white *Geotrichum*-like anamorphic state of *Bjerkander adusta*, sometimes incorrectly called *Polyporus adusta* (Barnett and Hunter Barry 1998). So far, only a few cases have been reported of isolating imperfect (anamorph) *Bjerkandera adusta* stage lacking sexual forms (Korniłłowicz-Kowalska et al. 2006a; Romero et al. 2007; Wirsel et al. 2001) and *Bjerkandera* sp. (Taboada – Puig et al. 2011). Korniłłowicz-Kowalska et al. (2006a) isolated anamorphic stage (*Geotrichum*-type sporulation) of *Bjerkandera adusta* from cultivated soil using pulp after industrial production of daunomycin as a substrate. The study showed (Korniłłowicz-Kowalska et al. 2006a) that *Bjerkandera adusta* strain CCBAS 930 had a unique ability to decolorize and biodegrade daunomycin, previously not described in fungi. In the literature, there is only information on the possibility of inactivation of structurally daunomycin-related anticancer drug—doxorubicin by actinomycetes *Streptomyces* WAC04685 (Westman et al. 2012). Daunomycin is an anthracycline antibiotic synthesized on an industrial scale with the participation of an *Actinobacterium*, *Streptomyces peucetius* (Grein 1987). This antibiotic and its conversion products are used as cytostatic drugs of a wide anticancer spectrum, especially in the treatment of lymphocytic leukemia.

*B. adusta* CCBAS 930 strain, as a basidiomycete anamorph of a white-rot fungus representing ligninolytic fungi, decolorizes and modifies many other colored aromatic compounds (Belcarz et al. 2005; Korniłłowicz-Kowalska et al. 2008; Korniłłowicz-Kowalska and Rybczyńska 2010, 2012, 2014). The decolorization of these compounds, similarly as the decolorization of daunomycin, is conditioned by the biosynthesis of oxidoreductases: peroxidases and/or laccases.

In assessing the applicability of microorganisms, in addition to determining the spectrum of decolorized color substances and degraded aromatic compounds, it is also important to know their nutritional demand with particular emphasis on C, N, and energy sources and the tolerance range for changes in the physical and chemical parameters of the environment due to the complex chemical nature of dyes wastewater, including color compounds, the content of acids or alkalis, and various salts.

In this context, this study, assigned to the assessment of the anamorphic *B. adusta* strain CCBAS 930 as a bioremediation agent, presents the results of research on the culture and physiological properties of this fungus and its tolerance to some physical and chemical parameters of the environment, including the selection of culture medium for biomass synthesis and the production of sporulating (idiophasic) aerial mycelium, determination of the spectrum of substrates utilized as C, N, and energy sources and related enzymatic activities as well as tolerance range for pH changes and osmotic pressure of the medium. Moreover, the specific ability of *B. adusta* CCBAS 930 for the degradation of daunomycin optimal conditions for decolorization and biodegradation of daunomycin in post-production effluents were determined.

## Materials and methods

### Fungal strain

Anamorphic *B. adusta* CCBAS 930 was isolated from the black earth soil (Pheozemes, FAO classification) as a strain decolorizing daunomycin, remaining in the post-production agarized *Streptomyces peucetius* mycelium, which is a waste of the pharmaceutical industry. The isolation procedure and strain identification are presented in the study by Korniłłowicz-Kowalska et al. (2006a). The characteristics of macro- and micromorphological features of the fungus are presented in the work by Korniłłowicz-Kowalska and Rybczyńska (2012).

### Culture condition

Experiments, subordinated to the general purpose of the research and detailed objectives were carried out in liquid cultures containing 50 cm^3^ of the appropriate medium. The homogenized mycelium of *B. adusta* strain CCBAS 930 (1 cm^3^) was the inoculum with a density of 10^5^ cfu obtained from a 7-day culture on a liquid glucose-potato medium. The control was a non-inoculated medium or medium inoculated with the fungus itself, depending on the purpose of the experiment. Fungus cultures and controls were incubated at 26 °C under stationary conditions. In some experiments, agitated cultures were also established. In all cases, 3 parallel culture replications were used.

### Selection of culture medium for the synthesis of biomass and idiophasic mycelium

The following media were tested:

- glucose-potato (PDA): extract 1 L, 200 g potato, 20 g glucose
- whey medium: whey 1 L, 10 g peptone, 5 g NaCl
- Sabouraud medium: 10 g pepton, 40 g glucose, 1 L H_2_O
- medium with maize extract: 1 L maize extract (from 40 g of maize flour)
- medium with asparagine (g L^−1^): l-asparagina 2.0, glucose 8.0, KH_2_PO_4_ 8.0; MgSO_4_ × 7H_2_O 0.5, FeCl_3_ 0.5, H_2_O 1 dcm^3^
- dextrose-mineral medium (g L^−1^): dextrose 0.1; NaCl 0.1; (NH_4_)_2_SO_4_ 0.1; KH_2_PO_4_ 0.1, biotin 0.0001, H_2_O 1 L
- Sanders medium (g L^−1^): KH_2_PO_4_ 0.2; K_2_HPO_4_ 0.15; NaH_2_PO_4_ 2.0; Na_2_HPO_4_ 1.5; NH_4_NO_3_ 0.6; NaNO_2_ 3.8; MgSO_4_ × 7H_2_O 0.3; 10 g cellulose (homogenized Whatman 1 filter paper); microelements (mg L^−1^): ZnSO_4_ × 7H_2_O 50.0; Fe_2_(SO_4_)_3_ × 6H_2_O 54.0; CuSO_4_ × 5H_2_O 2.5; MnSO_4_ 5.5; H_3_BO_3_ 5.7; H_2_O 1 L
- modified Sanders medium (10 g glucose instead of 10 g cellulose (homogenized Whatman 1 filter paper), remaining composition as above
- Czapek-dox medium (g L^−1^): NH_4_NO_3_ 2.0; K_2_HPO_4_ 1.0; MgSO_4_ 0.5; KCl 0.5; FeSO_4_ 0.01; sucrose 30.0; H_2_O 1 dcm^3^
- Czapek medium (g L^−1^): NaNO_3_ 2.0; K_2_HPO_4_ 1.0; MgSO_4_·7H_2_O 0.5; KCl 0.5; FeSO_4_·7H_2_O 0.01; sucrose 30.0; H_2_O 1 dcm^3^
- Park and Robinson medium (g L^−1^): NH_4_NO_3_ 0.1; K_2_HPO_4_ 0.2; MgSO_4_ × 7H_2_O − 0.5; glucose 0.7, H_2_O 1 L

The cultures were incubated for 14 days, after which the mycelium was drained and the dry weight of the mycelium was determined after drying at 105 °C. The transition from the phase of primary metabolism (trophophase) to secondary metabolism (idiophase) was also determined (macroscopically and microscopically) based on the moment of sporulating mycelium production.

### Analysis of the influence of incubation temperature, osmotic pressure, and medium pH

The optimum temperature for fungal growth was determined by measurements of linear growth and macroscopic observations of colony morphology on a glucose-potato agar medium (PDA) using a temperature of 5 °C, 20 °C, 25 °C, 30 °C, and 37 °C. The effects of pH and osmotic pressure of the medium were analyzed in stationary liquid cultures established on Park and Robinson medium with the initial pH in the range of 3.0–8.0 (pH was determined every 0.5 unit) and using NaCl at a concentration of 0.9, 3.0, 5.0, and 7.0%. The effect of pH with simultaneous indication of the optimum was determined on the basis of the increase in mycelium biomass assessed after 21 days of culture. The mycelium was drained and the dry mass determined at 105 °C. The ability to grow under different salinity conditions was determined by the macroscopic assessment of mycelium growth during 14-day incubation.

### Examination of physiological abilities and enzyme activities related to the fungal basic metabolism

Initial determination of physiological properties, which included the ability to hydrolyze complex organic substrates, i.e., starch, pectin, crystalline cellulose, tributyrin, chitin (colloidal solution), and gelatin was carried out on a mineral Park and Robinson solid medium with the addition of 1% of these substrates used as the only source C and energy (polysaccharides with the exception of cellulose and tributyrin) and the only source of C, N, and energy (gelatin, chitin). Media were inoculated with a mycelium disk of *ø* = 0.5 cm from a 7-day culture on Park and Robinson medium with glucose (cut off from the edge of the “colony”). Periodically, i.e., after 3, 7, and 14 days, macroscopic observations of fungal growth were carried out and the diameter of the decomposition zone was measured, observed directly as a transparent halo (hydrolysis of tributyrin and chitin), or visualized after pouring Lugol solution over the culture (starch hydrolysis), Frazier reagent (gelatin hydrolysis) (Korniłłowicz 1994) and 1% Merck N-cetyl-N <N <N-trimethylammonium bromide (pectin distribution) (Jayasankar and Graham 1970). Cellulolytic abilities were evaluated on the basis of mycelium growth on a Whatman 1 filter paper disc.

Determination of enzymatic activities was carried out in the case of a positive observation result regarding the tested physiological abilities in agar cultures. They included determination of proteolytic, amylolytic, pectinolytic, and lipolytic activities, and additionally, the activity against carboxymethylcellulose. Fungal cultures were carried out on liquid media using a clear culture fluid as the source of the enzyme. The control was inactivated post-culture fluid (by heating at 100 °C or by protein precipitation with trichloroacetic acid as for proteolytic activity determination). All enzymatic activities were determined by spectrophotometric methods (with the exception of lipolytic activity).

Proteolytic activity was determined in cultures on Park and Robinson medium with 1% gelatin as the sole source of C, N, and energy, using the modified Anson method (Korniłłowicz 1994) with 1% casein solution (Sigma), as a substrate in phosphate buffer at pH 7.6–7.8. The results were read from the standard curve prepared for tyrosine and expressed as μg of released tyrosine cm^−3^.

Amylolytic activity was determined in cultures on Park and Robinson medium with 2% starch (Sigma) as the only source of C and energy with a 2% starch solution in acetate buffer at pH 4.5 (Rogalski 1992). Amylolytic activity was determined based on the released glucose concentration determined by the method of Lloyd and Whelan (1969) using a glucose-oxidase reagent. The results were read from the standard curve prepared for glucose and expressed as μg of glucose cm^−3^.

Pectinolytic activity, including the activity of polygalacturonase (PG) and pectinase (PE), was determined in fungus cultures on Park and Robinson medium with 1% apple pectin as the only source of C and energy. Polygalacturonase (PG) activity was determined using a 1% polygalacturonic acid (ICN) in 0.05 M citrate buffer pH 4.5 (Rogalski 1992). The amount of released reducing sugars was determined by the Samogyi-Nelson method. The results were read from the standard curve prepared for glucose and expressed as μg of released reducing sugars cm^−3^. Pectinase (PE) activity was determined against 1% pectin solution (Fluca) in 0.05 M citrate buffer pH 4.5 (Rogalski 1992). The results were presented as above.

Lipolytic activity was determined in fungal cultures carried out on the medium described by Ota et al. (1968) containing (g dcm^−3^) 10 g olive oil, 10 g glucose, 4 g urea, 6 g KH_2_PO_4_, 1 g MgSO_4_ × 7H_2_O; 0.01 g FeCl 3 × 6H_2_O, 4 μg inositol, 8 μg biotin, 200 μg thiamine. Lipase activity was determined by the method of Sokolovska et al. (1998) using tributyrin (Sigma) in 0.05 M phosphate buffer pH 7.0. The enzymatic reaction was stopped and free fatty acids were extracted using a mixture of acetone and ethanol (1:1). The concentration of released fatty acids was measured by titration with 0.05 M KOH using phenolphthalein as an indicator. Nonspecific activity was expressed in enzymatic units (U), i.e., μmol of fatty acids released in 1 min, which is equivalent to 0.1 ml of KOH used. Specific activity was calculated per protein. The protein was determined by the Lowry et al.’s method (1954) using a standard curve prepared for bovine albumin.

### Analysis of simple phenol degrading abilities (methoxyphenols)

The cultures were grown on Park and Robinson (I) and Sanders medium, enriched with microelements (II) (“Analysis of the influence of incubation temperature, osmotic pressure, and medium pH” section) with the addition of 1% vanillic acid and syringic acid (Fluka) in a variant with 0.25% glucose and without glucose. The degree of utilization of the tested methoxyphenols was assessed on the basis of their content decrease, determined in post-culture fluids (A_500nm_), according to Malarczyk (1984), on the basis of a coupling reaction of phenols with sulfanilamide. The results were read from the standard curve prepared for vanillic acid and expressed as μg cm^−3^ of the medium.

### Analysis of alizarin degrading abilities

Initial determination of alizarin (1,2-hydroxyanthraquinone) degrading abilities was based on measurements of the discoloration zone on a solid Park and Robinson medium (“Analysis of the influence of incubation temperature, osmotic pressure, and medium pH” section) containing 2% of this dye. Liquid cultures were grown on Park and Robinson (I) medium and Sanders medium enriched with microelements (II) (“Analysis of the influence of incubation temperature, osmotic pressure, and medium pH” section) with the addition of 0.25% glucose and without glucose and then only on Park and Robinson medium with 0.25% glucose. Alizarin (Sigma-Aldrich) was prepared as a saturated solution in 0.1% NaOH and added to the medium at a concentration of 2%. Biodegradation of the dye was assessed based on the peroxidase activity and the size of the mycelial biomass. The activity of horseradish-type peroxidase (HRP-like) was assayed according to the method of Maehly and Chance (1954), modified by Malarczyk (1984), using 0.01% o-dianisidine (*ε*_460nm_ = 11.3 M^−1^ cm^−1^) as the substrate in 0.1 M acetate buffer, pH 5.5, in the presence of 0.1 mM H_2_O_2_. The mycelial biomass was determined using the weighing method by draining the mycelium at the end of the experiment and drying at 105 °C.

### Selection of optimal parameters of daunomycin decolorization

The preliminary experiment for the selection of the medium was carried out in liquid cultures of the fungus containing 10% and 20% of daunomycin effluents on media listed in the “Analysis of the influence of incubation temperature, osmotic pressure, and medium pH” section. The characteristics of the daunomycin post-industrial effluent are presented in the work by Korniłłowicz-Kowalska et al. (2006a). The remaining experiments were established on Park and Robinson medium with different doses of daunomycin (5, 10, and 30%). The effect of C (sugar) and nitrogen, pH, temperature, osmotic pressure, and aeration was analyzed.

The effect of sugars was studied on Park and Robinson medium with 30% daunomycin effluent and 0.25% fructose, glucose, lactose, maltose, or sucrose. The effect of the nitrogen source was tested on Park and Robinson medium without nitrogen and with 30% daunomycin effluent, as the sole source of C and energy, with the addition of 0.25% N used as (g dcm^−3^): NaNO_3_ 2.5 g; NH_4_NO_3_ 1.18 g; asparagine 2.0 g; urea 0.88 g; casein hydrolyzate. The effect of pH was tested on Park and Robinson medium with the addition of 10% daunomycin effluent as the sole source of C and N using pH in the range of 3.5–8.0 every 0.5 unit. The selection of the optimal temperature was analyzed by cultivating the fungus on solid Park and Robinson medium with 5% and 10% daunomycin effluent at 5 °C, 20 °C, 26 °C, 30 °C, and 37 °C using a mycelium disc of *ø* = 0.5 cm as inoculum. The effect of osmotic pressure was studied in the liquid medium of Park and Robinson with 30% effluent and addition of 0, 0.9, and 3.0% NaCl and aeration, on medium with 10, 20, and 30% effluent, in a water bath (26 °C) with agitation (120 rpm/min).

The decolorization rate of daunomycin was periodically estimated spectrometrically as Δ at 480 nm (maximal absorbance of daunomycin), in liquid cultures containing different concentrations of daunomycin effluent (Korniłłowicz-Kowalska et al. 2006a). The concentration of daunomycin was determined on the basis of a standard curve for a pure antibiotic (daunomycin, ICN). Macroscopic observations of the mycelium were carried out in parallel.

The optimal temperature was established by determining the shortest time of complete discoloration of the solid substrate with daunomycin. In parallel, the optima of the following physico-chemical parameters were determined: pH, osmotic pressure of the medium, and incubation temperature in cultures containing no daunomycin effluent. The same ranges of parameters and culture methods were used. The pH optimum was determined based on the size of the mycelium biomass in the liquid medium by measuring dry weight of mycelium at 105 °C. The influence of osmotic pressure was determined on the basis of macroscopic assessment of mycelial growth on a liquid substrate and the impact of temperature on the basis of colony diameter size and observation of macroscopic features of mycelium development on a solid medium (“Analysis of the influence of incubation temperature, osmotic pressure, and medium pH” section).

### Statistical analysis

Data are presented as means ± standard deviation (± SD) of three independent experiments. One-way ANOVA was used to quantitatively estimate the significance and relative contribution of medium composition, physico-chemical parameters to the overall decolorization, the activity of HRP-like peroxidase, phenols, and free radical contents. The data were analyzed via one-way analysis of variance (ANOVA) followed by Tukey multiple comparison test; *p* < 0.05 were considered significant

## Results

### Selection of culture media and growth conditions of *B. adusta* CCBAS 930

The growth rate of mycelium biomass and the production of sporulating aerial mycelium were considered as the criteria for the selection of the culture medium. Of the 11 tested natural or synthetic culture media used in two variants each: solid and liquid, the fastest growth rate of vegetative mycelium, measured by colony diameter (solid substrates) and biomass growth (liquid media) was obtained in cultures containing natural components: potato extract and whey (Table 1).

**Table 1:** Testing of culture media and physiological abilities of strain *B. adusta* CCBAS930

	1	2	3	4	5	6	7	8	9	10	11
mg of mycelium dry matter in 14-day cultures	1444.6 (± 22.30)	1151.5 (± 33.10)	623.5 (± 42.40)	953.4 (± 21.10)	136.4 (± 18.10)	21.5 (± 4.00)	162.6 (± 19.70)	0	0	137.7 (± 16.50)	155.3 (± 18.80)
Degradation zone after 7 days of growth (in cm)	A	B		C	D		E	F		G	
	9	9		9	5		0	5		2*	

1–11, liquid medium respectively: glucose-potato (1), whey medium (2), medium with maize extract (3), Sabouraud medium (4), medium with asparagine (5), dextrose-mineral medium (6), Sanders medium with glucose (7), Sanders medium with cellulose (8), Czapek (9), Czapek-Dox (10), Park and Robinson (11); Park and Robinson solid medium with the addition of starch (A), gelatin (B), tributyrin (C), pectin (D), cellulose (Whatman 1 filter paper disc) (E), carboxymethylcellulose (F), and/or chitin (G); *ns* not studied; * after 10 days

Production of sporulating aerial mycelium (*Geotrichum*-type conidia) was first observed on a mineral Park and Robinson medium with 0.07% glucose. The most intensive growth of sporulating aerial mycelium in liquid fungus cultures on this medium under different pH was obtained when the initial pH of the medium was 5.5–6.5, with a maximum at pH 6.0, with a possible increase in the pH range of 3.0–7.5 (Table 2). The optimal temperature of growth and synthesis of the sporulating aerial mycelium was 25–30 °C; after 7 days, it was 100% coverage of solid Park and Robinson medium (*ø* = 9 cm dishes). The temperature of 37 °C completely inhibited fungal growth. The temperature of 20 °C and lower slowed the rate of linear growth, and the effect was the strongest at 5 °C (Table 2).

**Table 2:** Optimal pH values of the medium and culture temperatures for growth and sporulation of *B. adusta* CCBAS930

Days of cultures 14	pH medium									
	3.0	4.0	4.5	5.0	5.5	6.0	6.5	7.0	7.5	8.0
	mg of mycelium dry matter									
	10.5 (± 2.12)	28.3 (± 4.10)	31.3 (± 4.40)	50.9 (± 5.10)	68.3 (± 5.70)	73.3 (± 7.60)	63.2 (± 7.00)	17.7 (± 2.50)	16.6 (± 3.10)	0
	Incubation temperature (°C)									
	5		20		25		30		37	
	Settlement of the substrate by mycelium spore (in %)									
3	0		25		50		50		0	
7	0		55		100		100		0	
10	20		65		100		100		0	
14	25		100		100		100		0	

### Examination of physiological abilities and enzyme activities related to the fungal basic metabolism

**Fig. 1:**
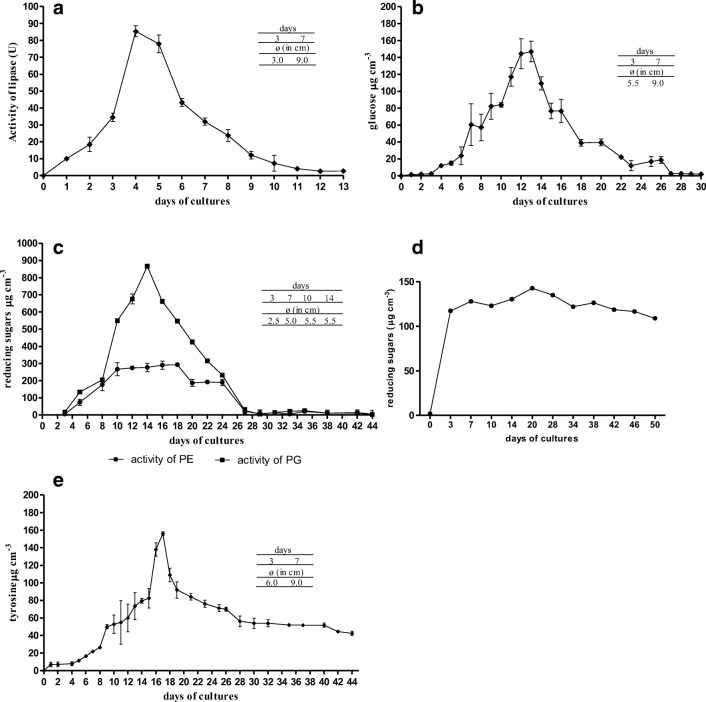
Activity of lipase (**a**) amylolytic (**b**), pectinolitic (**c**), celulolitic (**d**), and proteolytic (**e**) activity in liquid cultures of *B. adusta* CCBAS 930

It has been found that *B. adusta* strain CCBAS 930 hydrolyzes or cleaves 6 out of 7 tested complex organic substrates: starch, pectin, tributyrin, carboxylmethylcellulose (CMC), gelatine, and chitin. However, it does not degrade fibrous cellulose (filter paper). The tested strain showed the strongest degradative abilities, evaluated on the basis of the substrate decomposition zone in solid media, against triglycerides (tributyrin), followed by protein (gelatin) and starch. The smallest decomposition zone was recorded for pectin and colloidal chitin (for chitin, the values of days/*ø* in cm were 3/0, 7/2, 10/2, 14/2.5) (Table 1). Cultures on liquid media with the addition of the above substrates (except for colloidal chitin) as the sole source of C and energy or C, N, and energy confirmed preliminary observations (Fig. 1a–e). The test strain most efficiently hydrolyzed tributyrin, showing high lipase activities (Fig. 1a). It was noted that the highest activity of lipase, amylase, polygalacturonase (PG), pectinase (PE), carboxymethylcellulose, and proteases were marked at different times of culture and in a specific order. The maximum lipase production was visible the earliest, because on the 4th day of culture (Fig. 1a). In the second week of culture, the highest values were achieved by amylolytic and pectinic (PE) activities (Fig. 1b, c). The maximum production of carboxymethylcellulose and exoprotease was found the latest, since only in the 2nd half of the 3rd week of culture (Fig. 1d, e).

### Assessment of *B. adusta* CCBAS 930 ability to biodegrade simple aromatic compounds of natural origin

**Fig. 2:**
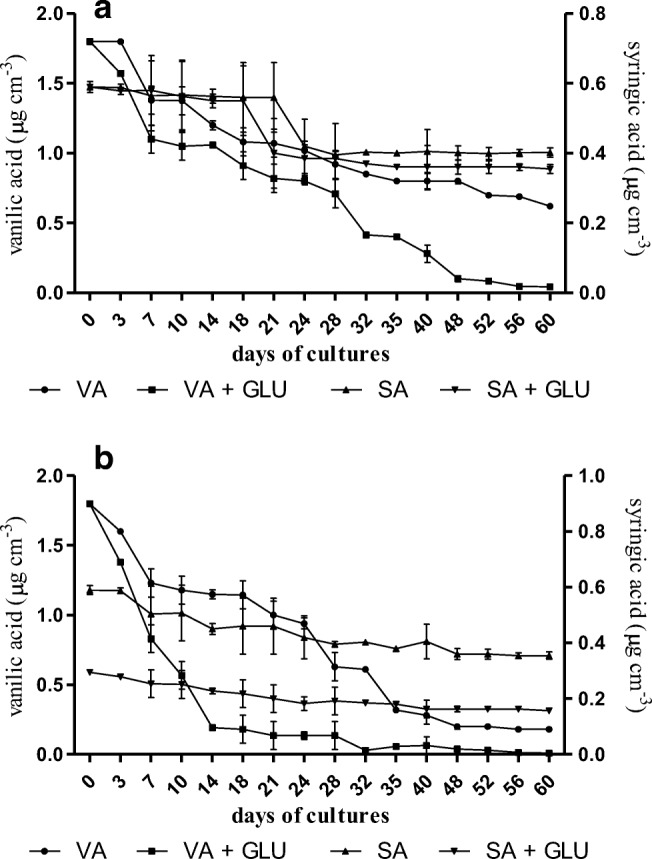
Content of methoxyphenol acids (VA vanilic acid, SA syringic acid) in cultures of *B. adusta* CCBAS 930 on Park and Robinson medium and supplemented with minerals with or without glucose (GLU)

It has been shown that *B. adusta* CCBAS 930 utilized simple phenols, such as vanillic acid and syringic acid as the only or additional (cometabolism) source of C and energy (Fig. 2a). The level of these methoxyphenols after 4 weeks of the fungus growth on Park and Robinson medium in the variant with glucose and without glucose, decreased by 50%, 34%, 60%, and 40%, respectively. The addition of microelements (Sanders medium with glucose) accelerated the rate of biodegradation of vanillic acid (94% degradation after 4 weeks of culture), but not of syringic acid (Fig. 2b).

### Assessment of *B. adusta* CCBAS 930 ability to biodegrade precursor of synthetic anthraquinone dyes—alizarin

**Fig. 3:**
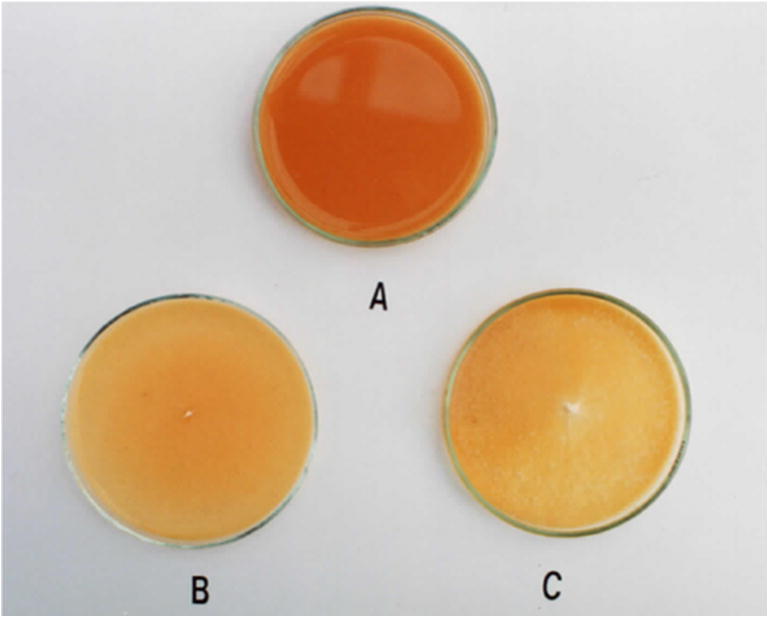
Decolorization of 2% alizarin in solid medium (**a**-control medium with 2% of alizarin, **b**, **c** decolorization of 2% alizarin in 18 days with *B. adusta* CCBAS 930)

**Fig. 4:**
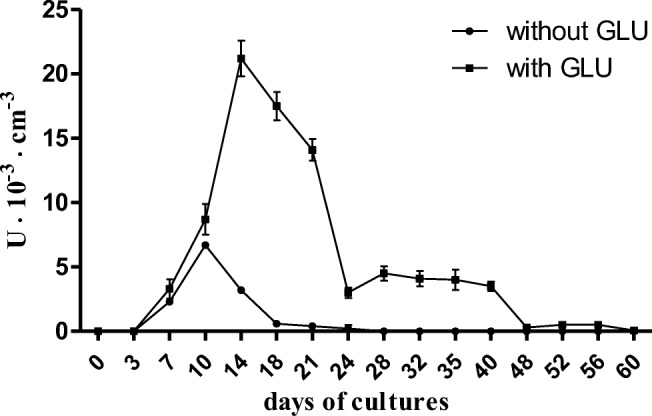
Activity of HRP-like peroxidases in cultures of *B. adusta* CCBAS 930 with 2% alizarin

It was shown that *B. adusta* strain CCBAS 930 was characterized by the ability to decolorize and biodegrade alizarin—a precursor in the synthesis of many synthetic anthraquinone dyes. Initial examination of abilities to biodegrade alizarin was conducted in solid cultures on Park and Robinson medium with glucose. The decolorization of the medium (alizarin is a yellow dye) became visible after 18 days (Fig. 3) of incubation and coincided with 100% colonization of the medium (Petri dish with *ø* = 9 cm) by sporulating aerial mycelium. Assuming peroxidase activity as an indicator of *B. adusta* CCBAS 930 activity against anthraquinone derivatives (Korniłłowicz-Kowalska et al. 2006a), it was found that the biodegradation of alizarin used at a concentration of 2% in stationary culture conditions, began on day 7, which was visible as a brightening of the culture fluid and was associated with the production of aerial mycelium (Fig. 3). Measurements of the intensity of medium color were not performed due to the cloudiness of the medium. On the other hand, it was found, based on the measurements of HRP-like peroxidase activity, that the test strain degraded alizarin in the presence of glucose (Fig. 4).

### Selection of optimal daunomycin biodegradation parameters in post-production effluent

#### Medium selection

Using daunomycin effluent decolorization rate as the evaluation criterion, Park and Robinson medium with glucose, Sanders with glucose and mineral with asparagine were considered as the most optimal ones (“Selection of culture medium for the synthesis of biomass and idiophasic mycelium” section). Complete decolorization of 10% daunomycin effluent was obtained the fastest in these media, i.e., after 7 days of fungus culture (Table 3). Park and Robinson medium was selected for further research, because of the simplest composition.

**Table 3:** Decolorization of daunomycin effluent by *B. adusta* strain CCBAS930 in different culture media

Complete decolorization of effluent (days) (concentration in %)	Medium										
	1	2	3	4	5	6	7	8	9	10	11
10	16 (± 0.16)	21 (± 0.20)	18 (± 0.14)	21 (± 0.10)	7 (± 0.11)	10 (± 0.14)	7 (± 0.12)	ns	10 (± 0.10)	10 (± 0.11)	7 (± 0.10)
20	21 (± 0.25)	21 (± 0.10)	21 (± 0.15)	21 (± 0.32)	10 (± 0.10)	14 (± 0.12)	10 (± 0.12)	ns	14 (± 0.10)	14 (± 0.12)	10 (± 0.12)

1–11 as in Table 1; *ns* not studied

#### Influence of carbon and nitrogen sources

Of the 5 sugars tested (mono- and disaccharides), the fastest decolorization effect of 30% daunomycin effluent, equivalent to the maximum reduction of daunomycin content (65% after 20 days of culture), was obtained in the presence of glucose (Table 4). It was expressed as over 40% degradation after 2 and after 3 weeks of incubation. Using 5 different mineral and organic nitrogen sources, the largest reduction in daunomycin content (65%) was obtained in the presence of nitrates. Based on these results, it can be assumed that the lack of nitrogen availability was a factor stimulating the biodegradation of daunomycin. This is confirmed by the research results on the use of a nitrogen source by the *B. adusta* CCBAS 930, which indicated that this strain does not grow in liquid Czapek medium with nitrates (= not utilize nitrates) (Table 1).

**Table 4:** Influence of various carbon and nitrogen sources on the rate of daunomycin degradation in the medium with 30% effluent

Medium	Days of cultures					
	0	14	17	20	24	28
	Concentration of daunomycin (in μg cm^−3^)					
Carbon sources						
Fructose	26.60 (0)*	25.29 (± 2.10) (5.0)**	25.19 (± 3.20) (5.30)	24.87 (± 3.20) (6.50)	22.1 (± 2.00) (6.9)	15.34 (± 1.05) (42.0)
Glucose	26.60 (0)	15.75 (± 1.15) (40.8)	10.75 (± 2.25) (59.6)	4.75 (± 0.90) (82.2)	3.50 (± 0.70) (86.8)	3.5 (± 0.70) (86.8)
Lactose	26.60 (0)	23.72 (± 2.35) (10.8)	14.02 (± 1.75) (43.0)	12.02 (± 2.50) (54.8)	10.01 (± 2.45) (62.4)	7.27 (± 1.20) (72.0)
Maltose	26.60 (0)	26.60 (0)	24.61 (± 1.95) (7.5)	23.50 (± 1.20) (16.7)	14.02 (± 0.80) (47.30)	13.44 (± 4.15) (49.50
Saccharose	26.60 (0)	26.60 (0)	26.60 (0)	25.08 (± 3.80) (7.7)	18.97 (± 3.20) (28.7)	9.75 (± 1.00) (63.34)
Nitrogen sources						
NaNO_3_	26.60 (0)*	23.45 (± 3.15) (11.9)**	21.80 (± 2.05) (18.0)	9.22 (± 0.85) (65.30)	5.16 (± 0.45) (80.6)	ns
NH_4_NO_3_	26.60 (0)	25.09 (± 4.20) (5.7)	23.80 (± 2.20) (10.5)	12.96 (± 1.35) (51.3)	10.75 (± 1.20) (59.6)	ns
Urea	26.60 (0)	26.24 (± 3.55) (1.4)	25.00 (± 3.35) (6.0)	10.38 (± 1.10) (60.9)	6.54 (± 0.25) (75.5)	ns
Asparagine	26.60 (0)	26.35 (± 3.00) (1.0)	24.70 (± 2.90) (7.1)	24.08 (± 2.60) (9.5)	13.07 (± 0.75) (59.9)	ns
Casein hydrolyzate	26.60 (0)	26.35 (± 4.25) (1.0)	23.90 (± 4.35) (10.2)	23.00 (± 2.00) (13.5)	16.86 (± 2.15) (36.6)	ns

*ns* not studied; * initial daunomycin content, ** decrease daunomycin content (in %) during treatment by *B. adusta* CCBAS 930

#### Influence of pH and osmotic pressure of the medium

It was found that the decolorization and biodegradation of daunomycin by the *B. adusta* strain CCBAS 930 was most efficient in the environment with pH 5.0–6.0, thus optimal conditions for the growth of this fungus and the transition to idiophase (production of sporulating aerial mycelium). Under these conditions, complete decolorization of 30% of daunomycin effluent was obtained after 7 days of culture (Table 5). Although the medium with pH 7.0–8.0 also rapidly decolorized 30% effluent, it was accompanied by the inhibition of fungal growth. The observed decolorization effect was most probably caused by daunomycin inactivation at higher pH, as reported by Reszka et al. (2005). It has also been shown that the process of daunomycin decolorization was the fastest in iso-osmotic conditions (0–0.9% NaCl), also optimal for the growth and development of this fungus. The increase in salt concentration (3% NaCl) inhibited the decolorization of 30% daunomycin effluent (Table 5).

**Table 5:** Influence of medium pH, osmotic pressure, and incubation temperature on decolorization of daunomycin effluent by *B. adusta* CCBAS930

Effluent concentrations (in %)	pH medium									
	3.5	4.0	4.5	5.0	5.5	6.0	6.5	7.0	7.5	8.0
	Complete decolorization (days of cultures)									
30	10 (++)	10 (++)	10 (++)	7 (+++)	7 (++++)	7 (++++)	7 (++)	7 (+)	7 (+)	7 (+)
NaCl (concentrations in %)										
30	0			0.9			3			
	−/3*, +/7, ++/7–14, +++/21, ++++/28			−/3, −/7, +/10–14, ++/21–28			−/3–28			
Incubation temperature (°C)										
	5	20		26		30		37		
Decolorization (in %)/days of cultures										
5	0	22/7; 56/14		22/4; 100/7		22/4; 100/7		0		
10	0	0/7; 50/14		0/4; 67/7; 100/14		67/7; 100/14		0		

(−) lack of growth; (+) weak growth of submerged mycelium, lack of decolorization, (++) strong growth of submerged mycelium, weak of aerial mycelium, lack of decolorization; (+++) strong growth of both submerged and aerial mycelium and visible decolorization of daunomycin (50% of decolorization), (++++) strong growth of both submerged and aerial mycelium and strong of decolorization (complete decolorization of daunomycin); * days of cultures

#### Influence of temperature and oxygenation of the culture

Conducting the *B. adusta* CCBAS 930 cultures on Park and Robinson medium with the addition of 5% and 10% daunomycin effluent in the temperature range from 5 to 37 °C, the fastest decolorization, after 7 and 14 days, respectively, was obtained at a temperature of 26–30 °C, and thus optimal for the growth of this fungus. Lower temperatures (20 °C) prolonged or inhibited (5 °C) daunomycin decolorization, while higher (37 °C) were mycotoxic (Table 5).

Aeration of the fungal culture in the medium with 10% and 20% daunomycin effluent by agitation inhibited daunomycin biodegradation. It caused sorption of this antibiotic by the mycelium. The effect of medium decolorization, as a result of biosorption was visible already after 7 (10% effluent) and 10 (20% effluent) days of submerged culture (Table 6). Comparably, this phenomenon has never been observed in stationary cultures, in which enzymatic daunomycin decolorization occurred in the medium.

**Table 6:** Decolorization of daunomycin effluents in liquid agitated cultures of *B. adusta* CCBAS 930

Effluent concentrations (in %)	Days of cultures					
	3	7	10	14	18	21
10	+	++	+++	+++	+++	+++
20	−	+	++	++	+++	+++

(−) lack of growth; (+) weak growth of mycelium, lack of decolorization, (++) visible growth of mycelium, 50% of decolorization; (+++) strong growth of mycelium and 100% of decolorization (biosorption)

## Discussion

The anamorphic white-rot fungus *Bjerkandera adusta* strain CCBAS930 is a mesophilic fungus with an optimum growth at 25–30 °C, growing at variable pH conditions (3.5–7.5) with an optimum in the range of 5.5–6.5 and salt concentration up to 3%. In terms of nutritional needs, it is a prototroph (simple sugar as a source of carbon and energy), characterized by a wide spectrum of complex C source and N-organic utilization, which includes various polysaccharides, lipids, solubilized lignin, protein, and chitin. It hydrolyzes lipids most effectively, chitin the least efficiently. It does not degrade, unlike the perfect stages of this fungus (white-rot fungus), crystalline cellulose (filter paper). The abilities to degrade many different organic polymers, as C, N, and energy sources, are a desirable property of the test strain, from the point of view of bioremediation. Many types of colored industrial wastewater, such as sewage of pulp and paper industry, some pharmaceutical and agri-food industries, e.g., sugar wastes, in addition to colored pollutants of aromatic structure (lignin, melanoids, anthracyclines, anthraquinone dyes), are loaded with an easily available organic substance (simple and complex carbohydrates, lipids, proteins) (Correia et al. 1994; Karlsson et al. 2001; Pokhrel and Viraraghavan 2004). These substrates allow the fungus to survive in the wastewater environment and biosynthesize mycelium, which produces extracellular degrading enzymes that break down aromatic contaminants. This is confirmed by the observations of growth and decolorization of raw post-production effluents containing daunomycin by *B. adusta* CCBAS930 (Korniłłowicz-Kowalska et al. 2006a, b). This study found the “succession” of the production of extracellular degradation enzymes of the tested strain. The maximum production of lipolytic enzymes became visible the earliest among them, because already after 4 days of culture, and the maximum production of proteolytic enzymes was recorded at the latest, after 3 weeks. Rapid biosynthesis of lipolytic enzymes may be related to the adaptation of the fungus to the chemical structure of plant residue colonized in the soil environment. The plant remains are covered with a cork layer (lignified debris) rich in lipid substances or a cuticle, under which there are tissues containing a lignin-cellulose complex. Lipid substances, such as resin acids, fatty acids, triglycerides, steryl esters, and sterols are also part of the wood resin. Ligninolytic fungi, when colonizing plant residues in the soil, must first cross the lipid barrier. The lipolytic activity of *Bjerkandera* sp. and *B. adusta* isolated from soil and leaf litter, and their relationship with lignin-cellulose degradation, was previously reported by Karlsson et al. (2001) and De Melo et al. (2018). Earlier studies on *Bjerkandera adusta* strain CCBAS930 (=*B. adusta* R59) showed (Belcarz et al. 2005) that lipase biosynthesis was also induced by the presence of humic acids isolated from brown coal, preparations of which contained lipids. The study of Ginalska et al. (2004) demonstrated that *B. adusta* CCBAS930 (denoted as *Geotrichum*-like R59) exhibited the maximum lipase production from different oils in a medium containing triglycerides, fatty acids, and triolein. It seems reasonable to propose this strain as a candidate for removal of triglycerides and other lipid components released in the paper making process due to the high activity of *B. adusta* CCBAS930 lipase against lipid substances present in wood and thermostability of this enzyme (Ginalska et al. 2004). These compounds are a serious problem in the pulp and paper industry and are traditionally removed by physical and chemical methods, but can also be dissolved biotechnologically (Farrell et al. 1997). The relatively late appearance of the proteolytic activity of *B. adusta* strain CCBAS930 poses some interpretation difficulties. It seems that this property on the one hand may be related to obtaining organic nitrogen from plant material due to the depletion of mineral nitrogen sources from the soil, e.g., N-NH_4_. It is known that extracellular proteinases of the fungus appear under the conditions of limited availability of the nitrogen source (Korniłłowicz-Kowalska 1999). On the other hand, it cannot be ruled out that this may be coupled with the transition of the fungus from the vegetative phase to the fungal sporulation phase. Leonowicz et al. (2001) emphasized that the growth of fungi decomposing wood, especially in natural conditions, requires “control of their nitrogen metabolism.” The “late maxima” of proteolytic activity of the fungus may be conditioned by the involvement of proteinases in mycelium differentiation processes (sporulation) and the functioning of ligninolytic enzymes due to the fact that the process of ligninolysis in white-rot Basidiomycetes is a secondary metabolism process. As our previous research has shown (Korniłłowicz-Kowalska et al. 2008), the production of sporulating aerial mycelium and maxima of peroxidase activity are synchronized with each other. They appear in the third week of fungus culture on a medium with lignin. Therefore, they coincide with the formation of fungal proteolytic activity observed in this work. The above assumption is based on the results of the study of Staszczak et al. (2000), who provided proof of the non-nutritional and non-morphogenetic function of proteinases in white-rot fungi. According to the cited authors, proteinases of white-rot fungus, *Trametes versicolor*, are involved in the regulation of HRP-like and laccase activities of this fungus.

The lack of ability of the anamorphic stage of *B. adusta* to hydrolyze cellulose fibers with the simultaneous use of soluble cellulose (carboxymethylcellulose) deserves a special mention. This fact from a practical point of view can be used for the bleaching of cellulose fibers, e.g., cotton. Cotton is mainly composed of crystalline cellulose and contains certain amounts of non-cellulosic ingredients, including colored ones, which must be removed from the fibers in the production process. Cotton bleaching effluent (Zhang et al. 1999) is brown, as are the effluents from wood pulp bleaching. Raw enzyme preparations containing HRP-like of anamorphic *B. adusta* strain CCBAS930 can therefore be considered as an alternative to the physico-chemical methods of bleaching cellulose fibers.

The preference for acidic environments by *B. adusta* CCBAS930 predisposes this strain for the decolorization of low pH industrial waste, such as daunomycin and post-dye industry effluents, containing anthraquinone dyes and triarylmethane dyes, used in protein fiber dyeing: wool and silk (Ghaly et al. 2014; Korniłłowicz-Kowalska et al. 2006a).

Studies of decolorization and biodegradation activity of *B. adusta* CCBAS930 conducted in this and earlier works (Korniłłowicz-Kowalska et al. 2006a; Korniłłowicz-Kowalska and Iglik 2008; Korniłłowicz-Kowalska and Rybczyńska 2012, 2014) showed that these properties concerned at least 13 different types of colored contaminants with aromatic structure occurring in industrial wastewater, which includes various solubilized lignin fractions (black liquor, post-vanillin lignin), anthracyclines, such as daunomycin and structurally related anthraquinone dyes (Alizarin Remazol Brillat Blue R, Carmine Acid, Poly R-478), triarylmethane dyes (Brillant Green), xanten dyes, (Erythrosine), humic acids of various origins (from brown coal, chernozem and lessive soil), and melanoids (unpublished data) causing coloration of sugar waste and molasses. Our study indicated that *B. adusta* CCBAS strain also degrades simple phenols with one (vanillic acid) or two (syringic acid) methoxyl groups. The current study demonstrates that this strain relatively quickly, i.e., within 7 days, decolorizes enzymatically 3× diluted post-production daunomycin effluents with a concentration of 30 μg cm^−3^.

The screening of *B. adusta* CCBAS930 culture media carried out in this work indicates that media containing natural components, such as potato extract and whey are suitable for the purpose of obtaining large amounts of mycelium biomass, e.g., for the purpose of biosorption processes. The present study and the work of Korniłłowicz-Kowalska et al. (2008) showed that *B. adusta* strain CCBAS930 exhibited sorption properties only in submerged culture conditions when it did not form aerial mycelium. Novotný et al. (2004) found that the biosynthesis of ligninolytic enzymes, i.e., peroxidase and laccase, was inhibited in the submerged culture of white-rot fungus. It seems that *B. adusta* biomass can be used primarily for the decolorization by sorption of concentrated effluents, such as black liquor. The successful use of biomass of another white-rot fungus, *Steccherinum* sp., for the decolorization of black liquor by biosorption was reported by Da Re and Papinutti (2011). We believe that the brown-colored mycelium obtained after the biosorption process as a by-product of the decolorization process can be successfully processed into compost, after mixing with vegetable waste rich in easily digestible organic matter, e.g., green waste. Large quantities of polyphenols adsorbed by *B. adusta* CCBAS930 mycelium may be used as a precursor of compost humic acids in the composting process, which is an additional, valuable component of this biomass.

Liquid media poor in nutrients with one simple organic C source and a low nitrogen content, and stationary growth conditions were found to be the most optimal substrates for the purposes of rapid transition from the vegetative phase to the sporulation phase (=production of aerial mycelium), morphological equivalent of the fungal secondary metabolism. The fastest induction of aerial mycelium was obtained in a liquid medium of Park and Robinson with 0.07% glucose and 0.015% NH_4_NO_3_. The enzymatic (in the medium) decolorization of daunomycin was also most efficient in the above medium as well as in two other media with glucose and low concentration of N mineral (Table 4). As we have demonstrated in our previous works, the process of daunomycin biodegradation by *B. adusta* CCBAS930 is a cometabolic process requiring an additional source of C and energy, such as glucose (Korniłłowicz-Kowalska et al. 2006a). The fact that decolorization of daunomycin effluent also rapidly occurred in stationary cultures on the Czapek medium containing nitrates—an unabsorbable nitrogen source by *B. adusta* CCBAS930—demonstrated the importance of nitrogen deficit in the induction of secondary metabolism of this fungus and associated peroxidase biosynthesis. Similar observations were reported earlier by Robinson et al. (2001), who showed that *B. adusta* in cultures with RBBR synthesized peroxidases (LiP, MnP) and laccase under deficiency of nitrogen mineral salts. The cause of this phenomenon was explained by Kirk et al. (1978), who showed that the biosynthesis of ligninolytic enzymes, such as peroxidase is induced by nitrogen starvation. The stimulation of the production of ligninolytic enzymes by white-rot fungi also requires static culture conditions that enable the transition to the sporulation phase and the corresponding secondary metabolism phase (Novotný et al. 2004).

In light of our results, it seems most feasible for industrial practice to use this anamorphic fungal strain for enzymatic decolorization of effluents generated during daunomycin production. This strain grows and degrades daunomycin not only in diluted, but also after adaptation in raw (Korniłłowicz-Kowalska et al. 2006a), highly toxic post-production effluents. Our work demonstrated that *B. adusta* strain CCBAS930 most effectively degraded daunomycin in stationary cultures with 0.25 g dm^−3^ glucose, medium pH of 5.0–6.0 and 0.7% salt concentration (NaCl), and culture temperature of 26–30 °C. Similar pH values (5.0–6.0) were adopted as the optima for peroxidase activity of *B. adusta* by Moreira et al. (1997) (Mn-dependent peroxidase) and Anastasi et al. (2011) (Mn-dependent and versatile VP peroxidase). The strong affinity of *B. adusta* CCBAS930 for anthraquinone dyes also indicated real possibilities of using this fungus in such biotechnological processes as decolorization of effluents from wool dyeing processes and other protein fibers stained with anthraquinone dyes (Korniłłowicz-Kowalska and Rybczyńska 2010, 2014).

Our own research shows that *B. adusta* CCBAS930 biodegradation of low molecular weight aromatic contaminants containing anthraquinone groups (anthracyclines, alizarine) with the involvement of horseradish-like peroxidase (HRP-like) includes demethylation reactions (reduction of the level of methoxyphenols) and generation of free radicals, accompanied by a loss of color. Demethylation is a process that plays a fundamental role in lignin depolymerization (Leonowicz et al. 2001). Our research carried out in the present and earlier works (Korniłłowicz-Kowalska et al. 2006a, 2008; Korniłłowicz-Kowalska and Rybczyńska 2014) has shown that the demethylation reaction occurs during *B. adusta* CCBAS930 biodegradation of lignin, humic acids, daunomycin, and anthraquinone dyes. Free radicals are also released during transformations of all these anthraquinone derivatives in the cultures of the tested fungus. According to Leonowicz et al. (1991), the released smaller subunits are converted in free radical transformations into phenols and quinones during the biodegradation of aromatic polymers, such as lignin by extracellular oxidation enzymes. HRP-like peroxidase is a new enzyme of *B. adusta* fungus catalyzing the biodegradation of post-industrial lignin and related anthraquinone derivatives, i.e., daunomycin, anthraquinone dyes and humic acids. Due to the specific preferences of *B. adusta* CCBAS930 towards anthraquinone derivatives, this enzyme is the most similar to dye-decolorizing peroxidase (DYP) of *Bjerkandera adusta* Dec1. Dye-decolorizing peroxidase (Kim and Shoda 1999; Sugano et al. 2006, 2009; Sugano 2009; Yoshida et al. 2011) degrades not only typical peroxidase substrates, but also hydroxyl-free antraquinones, which are not substrates of other peroxidases.

The research carried out in this work shows that biodegradation of simple phenolic acids—lignin monomers, i.e., vanillic and syringic acids, may also occur without the participation of an additional carbon source. This demonstrates the ability of *B. adusta* CCBAS930 enzymes to break down the aromatic structure of some compounds, such as simple methoxyphenols and utilize them as a source of C and energy. The decrease in the content of phenols from the OCH_3_ group in the experiments with vanillic and syringic acids as the only source of C and energy, with simultanous mycelium biosynthesis, indicated that demethylation led to dearomatization and the inclusion of the resulting products in the fungal basic metabolism. The fact that demethylation led to the breaking of the ring was reported by Paździoch-Czochra et al. (2003). The observed property increases the application value of *B. adusta* strain CCBAS930, because both tested phenolic compounds are present in the waste water of numerous industries, including pulp and paper industry. Biodegradation of alizarin, i.e., dihydroxyanthraquinone in cultures of *B. adusta* CCBAS930 synthesizing HRP-like, probably occurs by oxidation. This assumption is based on the studies of Arrieta – Baez et al. (2002), who reported that horseradish peroxidase (HRR) catalyzes alizarin oxidation. In turn, Reszka et al. (2005) showed that horseradish peroxidase can oxidize hydroquinone in an anthracycline molecule, generating free radicals (phenoxyl radical) and causing degradation of this cytostatic drug.

Previous studies concerning degradation abilities of *B. adusta* strain CCBAS930 primarily indicated the capability of this fungus to biodegrade anthraquinone derivatives. This differentiates this strain from other strains of this species (Heinfling et al. 1998; Moreira et al. 1997). Taking this into account, the current study also identified some factors affecting the decolorization and degradation of daunomycin, the most important anthraquinone derivative degraded by this fungus.

It was shown that the biodegradation of daunomycin in post-production effluents occurs most effectively in a mineral medium with glucose, with a low form of nitrogen (N-NH_4_) available for this strain or containing N-NO_3_—a form of nitrogen that is not assimilated by this strain. In addition, daunomycin decolorization required the presence of phosphates and magnesium ions. The remaining parameters of the medium, i.e., pH and osmotic pressure as well as the temperature of the culture corresponded to the optimal growth conditions of *B. adusta* strain CCBAS930. The indicated parameters are mostly consistent with the data of other authors (Radha et al. 2005; Singh et al. 2015) regarding the assessment of factors affecting the growth and regulating the decolorization and biodegradation of anthraquinone derivatives, i.e., mono- and polyantraquinone dyes. Our investigations demonstrated that HRP-like biosynthesis by *B. adusta* strain CCBAS930 occurred in nitrogen-deficient media. The preferred source of nitrogen is N-NH_4_, while N-organic is not conducive to decolorization. The optimum pH for the process of decolorization and biodegradation of daunomycin by *B. adusta* CCBAS930 was found to be 5.0–6.0. This is a higher value in relation to the pH of most industrial effluents containing anthraquinone derivatives (anthracyclines and anthraquinone dyes), where the pH is in the range of 3.5–4.8 (Bisschop and Spanjers 2003; Ghaly et al. 2014; Korniłłowicz-Kowalska et al. 2006a). It is also higher in relation to the pH optimum for the maximum decolorization of anthraquinone dyes by other white-rot fungi, such as *P. chrysosporium*, where this process was most effective at pH 4.0–5.0, and at pH < 4.0 and > 5.0, it decreased (Radha et al. 2005). The research of other authors (Anastasi et al. 2011) showed, however, that the optimal pH for the decolorization of some anthraquinone dyes was 5.0–6.0. The optimal temperature for growth and synthesis of daunomycin decolorizing enzymes in *B. adusta* CCBAS930 cultures was 25–30 °C. This is the optimal temperature for most mesophilic white-rot fungi (Singh et al. 2015) However, the agitation of the culture was not conducive to the enzymatic decolorization of daunomycin by *B. adusta*. Although process of biodegradation of lignin and its derivatives by white-rot fungi is an aerobic process, oxygenation of the culture by agitation only caused sorption of daunomycin by the mycelium. Kirk et al. (1978) were one of the first one who reported the inhibitory effect of *P. chrysosporium* culture agitation on lignin degradation. We believe that maintaining the culture in the trophophase (vegetative phase) and inhibition of the transition to idiophase (reproduction phase), which corresponds to the secondary metabolism and HRP-like peroxidase induction associated with it, are the main reasons for daunomycin biodegradation inhibition in agitated cultures of *B. adusta* CCBAS930.
